# Changes in the central component of the hypothalamus-pituitary-thyroid axis in a rabbit model of prolonged critical illness

**DOI:** 10.1186/cc8043

**Published:** 2009-09-11

**Authors:** Liese Mebis, Yves Debaveye, Björn Ellger, Sarah Derde, Eric-Jan Ververs, Lies Langouche, Veerle M Darras, Eric Fliers, Theo J Visser, Greet Van den Berghe

**Affiliations:** 1 Department of Intensive Care Medicine, Katholieke Universiteit Leuven, Herestraat 49, Leuven, B-3000, Belgium; 2 Laboratory of Comparative Endocrinology, Katholieke Universiteit Leuven, Naamsestraat 61, Leuven, B-3000, Belgium; 3 Department of Endocrinology and Metabolism, Academic Medical Center, University of Amsterdam, Meibergdreef 9, Amsterdam, 1105 AZ, The Netherlands; 4 Department of Internal Medicine, Erasmus University Medical Center, Dr. Molewaterplein 50, Rotterdam, 3015 GE, The Netherlands

## Abstract

**Introduction:**

Prolonged critically ill patients reveal low circulating thyroid hormone levels without a rise in thyroid stimulating hormone (TSH). This condition is labeled "low 3,5,3'-tri-iodothyronine (T_3_) syndrome" or "nonthyroidal illness syndrome (NTI)" or "euthyroid sick syndrome". Despite the low circulating and peripheral tissue thyroid hormone levels, thyrotropin releasing hormone (TRH) expression in the hypothalamus is reduced and it remains unclear which mechanism is responsible. We set out to study whether increased hypothalamic T_3 _availability could reflect local thyrotoxicosis and explain feedback inhibition-induced suppression of the TRH gene in the context of the low T_3 _syndrome in prolonged critical illness.

**Methods:**

Healthy rabbits were compared with prolonged critically ill, parenterally fed animals. We visualized TRH mRNA in the hypothalamus by in situ-hybridization and measured mRNA levels for the type II iodothyronine diodinase (D2), the thyroid hormone transporters monocarboxylate transporter (MCT) 8, MCT10 and organic anion co-transporting polypeptide 1C1 (OATP1C1) and the thyroid hormone receptors α (TRα) and β (TRβ) in the hypothalamus. We also measured the activity of the D2 and type III iodothyronine deiodinase (D3) enzymes.

**Results:**

In the hypothalamus of prolonged critically ill rabbits with low circulating T3 and TSH, we observed decreased TRH mRNA, increased D2 mRNA and increased MCT10 and OATP1C1 mRNA while MCT8 gene expression was unaltered as compared with healthy controls. This coincided with low hypothalamic thyroxine (T_4_) and low-normal T_3 _concentrations, without a change at the thyroid hormone receptor level.

**Conclusions:**

Although expression of D2 and of the thyroid hormone transporters MCT10 and OATP1C1 were increased in the hypothalamus of prolonged critical ill animals, hypothalamic T_4 _and T_3 _content or thyroid hormone receptor expression were not elevated. Hence, decreased TRH gene expression, and hereby low TSH and T3 during prolonged critical illness, is not exclusively brought about by hypothalamic thyrotoxicosis, and infer other TRH suppressing factors to play a role.

## Introduction

Prolonged critically ill patients reveal a suppressed neuroendocrine function with low circulating levels of several anterior pituitary-dependent hormones [1]. The severity of these neuroendocrine alterations was shown to be related to adverse outcome of patients in the intensive care unit [1,2].

The thyroid axis is driven by thyrotropin releasing hormone (TRH) from the paraventricular nucleus (PVN) of the hypothalamus. TRH stimulates the release of thyroid stimulating hormone or thyrotropin (TSH) from the pituitary, which in turn drives the thyroid gland to produce and release the prohormone thyroxine (T_4_) and to a minor extent the active hormone 3,5,3'-triiodothyronine (T_3_). T_4 _is metabolized in peripheral tissues to produce T_3_. There is a typical negative feedback regulation from T_3 _and T_4 _at the level of the pituitary and the hypothalamus. During prolonged critical illness, circulating T_3 _levels are low and in severe and prolonged cases, T_4 _levels are also reduced [3]. This condition is referred to as the 'low T_3 _syndrome', the 'non-thyroidal illness syndrome' or the 'euthyroid sick syndrome', different names that reflect the uncertainty regarding its origin and clinical implications. Despite the low levels of circulating and peripheral tissue thyroid hormones, TRH expression in the hypothalamus is reduced [4] and the TSH secretory pattern shows a dramatic loss in pulsatility, which correlates positively with the low serum T_3 _concentrations [1,3]. The reduced TSH release seems to be secondary to the diminished drive by TRH [1]. It remains unclear which mechanism is responsible for the reduced hypothalamic TRH expression during prolonged critical illness.

Several mechanisms have been proposed for the suppression of the hypothalamus-pituitary-thyroid (HPT) axis during critical illness, among which is a local thyrotoxicosis in the hypothalamus. Increased hypothalamic T_3 _availability could indeed explain feedback inhibition-induced suppression of the TRH gene in the context of the low T_3 _syndrome. A first mechanism for increasing the local concentration of T_3 _in the hypothalamus is increased local conversion of T_4 _to T_3_. More than 80% of T_3 _in the brain originates from local T_4 _to T_3_conversion by the type II iodothyronine deiodinase (D2) [5]. Therefore, an upregulation of D2 in the mediobasal hypothalamus could lead to a local hyperthyroid state which in turn would suppress TRH in hypophysiotropic neurons. Injection of lipopolysacharide in rats and mice has been shown to upregulate hypothalamic D2 expression and activity [6-9]. Alternatively, decreased inactivation of T_3 _and T_4 _by the type III iodothyronine deiodinase (D3) could also lead to higher hypothalamic thyroid hormone levels suppressing TRH. In line with this, a mouse model for chronic inflammation showed decreased D3 mRNA expression in the region of the hypothalamic PVN [10].

A second possible mechanism by which local iodothyronine levels in the hypothalamus could be increased is elevated transport of iodothyronines into the hypothalamus. The entry of thyroid hormone from the circulation into the hypothalamus is mediated by specific thyroid hormone transporters of which two categories have been identified, organic anion transporters and amino acid transporters. Na^+^-independent organic anion co-transporting polypeptides (OATPs) represent a large family of homologous proteins of which OATP1C1 (SLCO1C1) shows a high specificity and affinity towards iodothyronines, in particular T_4 _and reverse T3 (rT_3_) [11,12]. OATP1C1 is mainly expressed in brain capillaries and is considered to be important for the uptake of T_4 _across the blood-brain barrier [11-13]. The human monocarboxylate transporter 8 (MCT8), a specific thyroid hormone transporter, is also expressed in the hypothalamus and transports T_4 _and T_3 _in a Na^+^-independent manner [14]. Study of MCT8 null-mice suggests that its expression is necessary for normal feedback regulation of TRH neurons in the hypothalamus [15,16]. MCT10 was identified as a T-type amino-acid transporter [17,18] and was recently shown to be at least as active for thyroid hormone transport as MCT8 [19]. The role of these transporters in hypothalamic feedback regulation in critically ill patients is currently unknown.

The major effects of thyroid hormone are exerted by interaction with its nuclear receptors. Thereby, a third mechanism explaining a lower TRH expression in the face of normal or low thyroid hormone levels could be an increased activity of the available thyroid hormone by increasing the expression of the nuclear thyroid hormone receptors. No data on hypothalamic expression of the different thyroid hormone receptors in prolonged critical illness are currently available.

Our goal was to study if increased local T_3 _content in the hypothalamus, brought about by these different potential mechanisms, suppresses hypophysiotropic TRH neurons during prolonged critical illness.

## Materials and methods

### *In vivo *animal experiment

All animals were treated according to the Principles of Laboratory Animal Care formulated by the U.S. National Society for Medical Research and the Guide for the Care and Use of Laboratory Animals prepared by the National Institutes of Health. The study protocol was approved by the Leuven University ethical review board for animal research (P03052).

The model has been described in detail previously and is shown to reproduce the bi-phasic response to critical illness as seen in the human situation [20,21].

Male New Zealand White rabbits were housed individually and exposed to artificial light for 14 hours per day. On day 1, rabbits were anesthetized with 30 mg/kg ketamine, intramuscularly (Merial, Lyon, France), and 0.15 mg/kg medetomidine, intramuscularly (Orion, Ospoo, Finland). Their neck and flanks were shaved and anesthesia was than supplemented by isoflurane (Isoba Vet.; Schering-Plough, Brussels, Belgium) added to the breathing gas via regular vaporizer. Thereafter, a supplemental local paravertebral block with xylocaine 1% (Astra Pharmaceuticals, Brussels, Belgium) was performed and a full-thickness burn injury equaling 15 to 20% total body surface area was imposed. At the end of the procedure, animals returned to their cages where overnight fluid resuscitation was started with a continuous infusion of Ringer's lactate at six drops per minute (± 18 ml/h) through a volumetric infusion pump (IVAC 531 infusion pump, IVAC cooperation, San Diego, CA, USA). All animals received parenteral nutrition (PN) from day 2 onwards to avoid starvation-induced endocrine alterations. The PN infusion bags contained 150 ml of Clinomel N7 (Baxter, Clintec Parentéral, Maurepas, France) and 175 ml sterile water. Thus, bags with 325 ml solution contained 156 kcal non-protein calories and 0.99 g nitrogen. Of all non-protein calories, 61.5% were delivered as carbohydrates, and 38.5% as fat. Protein intake equaled 1.49 g/kg per day. No additional vitamins or trace elements were added. PN was infused at four drops a minute (± 12 ml/h). Animals had free access to water, but oral food intake was denied. Blood glucose levels were kept below 180 mg/dl by frequent blood glucose monitoring and titration of insulin infusion (100 IU/ml; Actrapid Novolet, Novo Nordisk, Bagsvaerd, Denmark; via an SE200B infusion pump, Vial Medical, Brezins, France) when necessary. In total, 25 animals survived 7 days of illness, after which blood was taken and serum was stored at -20°C. Animals were sacrificed and a tissue block containing the hypothalamus (rostral border just anterior of the optic chiasm, caudal border through the mamillary bodies, dorsal border through septum) was dissected and snap-frozen in liquid nitrogen. Healthy animals (n = 25), matched for gender, age, and body weight, that had free access to regular chow, were studied as controls.

### Serum analysis

Plasma concentrations of TSH were measured by a specific radioimmunoassay (RIA; reagents provided by Dr. A. Parlow, National Pituitary Agency). The detection limit was 1.2 mIU/l, and the intra assay coefficient of variation (CV) was 5.3%. In one sample from a prolonged ill rabbit TSH was below the detection limit. Total concentrations of plasma T_4 _and T_3 _were determined by an in-house RIA [22]. The detection limit and intra assay CV were, 5 and 2 fmol and 2.8% and 2.2%, respectively. No free hormone determinations were performed because blood sampling was performed with heparinized catheters which is known to artefactually alter the free fraction of thyroid hormones in the samples [23].

### D2 and D3 activity

The complete hypothalamic block of five control animals and six prolonged ill rabbits were homogenized on ice in 10 volumes of PED10 buffer (0.1 M phosphate, 2 mM EDTA, 10 mM DTT, pH 7.2) using a Polytron (Kinematica AG, Lucerne, Switzerland). Homogenates were cooled on ice and immediately analyzed. Protein concentration was measured with the Bio-Rad Protein Assay (Bio-Rad, Veenendaal, The Netherlands) using BSA as the standard following the manufacturer's instructions. D2 and D3 activities were assayed [24] by duplicate incubations of homogenate (final protein concentration about 4 mg/ml) for 60 minutes at 37°C with 1 nM [3',5'-^125^I]T_4 _(200,000 cpm) in a final volume of 0.1 ml PED10 buffer. The incubations were carried out in the presence of 0.1 mM propylthiouracil to inhibit possible D1 activity, and in the absence or presence of 0.1 μM T3 to saturate D3 activity. Reactions were stopped by addition of 0.1 ml 100% methanol on ice. After centrifugation, 0.1 ml of the supernatant was added to 0.1 ml 0.02 M ammonium acetate (pH 4.0), and 0.1 ml of the mixture was applied to a 4.6 × 250 mm Symmetry C18 column connected to an Alliance high-performance liquid chromatography system (Waters, Etten-Leur, The Netherlands). The column was eluted with a linear gradient of acetonitrile (28 to 42% in 15 minutes) in 0.02 M ammonium acetate (pH 4.0) at a flow of 1.2 ml/min. The radio-activity in the eluate was measured on-line using a Radiomatic A-500 flow scintillation detector (Packard, Meriden, CT, USA). Activity eluting in the T_3 _and rT_3 _fractions represented D2 and D3 activity, respectively.

### Thyroid hormone concentration in the hypothalamus

We used the hypothalamic block of 10 healthy rabbits and 11 prolonged critically ill rabbits to measure T_4 _and T_3 _content. Iodothyronines were extracted and purified from the hypothalamus and T_4 _and T_3 _were measured by RIA, as described previously [25,26]. In brief, the entire hypothalamic block was homogenized directly in methanol, and 2000 cpm of outer ring labeled [^131^I]T_4 _and [^125^I]T_3 _were added to each sample as internal tracers for recovery. Appropriate volumes of chloroform were added to extract with chloroform/methanol (2:1) twice. The iodothyronines were then back-extracted into an aqueous phase and purified by passing this aqueous phase through Bio-Rad AG 1 × 2 resin columns (Bio-Rad, Veenendaal, The Netherlands). After a pH gradient, the iodothyronines were eluted with 70% acetic acid, evaporated to dryness, and resuspended in RIA buffer. The extracts were counted to determine the recovery of [^131^I]T_4 _and [^125^I]T_3 _added to each sample. For the present experiment the average recovery was 50.5% for [^131^I]T_4_, and 74.5% for [^125^I]T_3_. Concentrations were calculated using the amounts of T_4 _and T_3 _found in the respective RIAs, the individual recovery of [^131^I]T_4 _and [^125^I]T_3 _added to each sample, and the weight of the tissue sample submitted for extraction.

### Cloning of rabbit genes

Total RNA was isolated from rabbit hypothalamic tissue using Qiazol lysis reagent (Qiagen, Venlo, The Netherlands) and subsequently purified using the RNeasy mini RNA isolation kit (Qiagen, Venlo, The Netherlands). cDNA was obtained by reverse transcription of 2 μg total RNA using random hexamer primers. Oligonucleotides homologous to sequences surrounding the start or stop codons of human, mouse and rat proTRH, D2, MCT8, MCT10, OATP1C1, thyroid hormone receptor (TR) α1, TRα2, TRβ1 and TRβ2 were designed and used for PCR. The amplified fragments were cloned into the pGEM-T vector followed by sequence analysis. These sequences showed high amino acid identity with the corresponding genes from other mammalian species and data have been submitted to the GenBank database: proTRH [GenBank:EF370408], D2 [GenBank:EU489480], MCT8 [GenBank:EF420874], MCT10 [GenBank:EF489851], OATP1C1 [GenBank:EF420875], TRα1 [GenBank:EU489476], TRα2 [GenBank:EU489477], TRβ1 [GenBank:EU489478] and TRβ2 [GenBank:EU489479]. Based on these sequences, we designed specific primers and probes for real-time PCR analysis and in situ hybridization.

### Fluorescence *in situ *hybridization

TRH and D2 mRNA expression was analyzed in two healthy vs. two prolonged ill animals. The TRH and D2 *in situ *probes were generated in our laboratory. The TRH probe was complementary to 1 to 203 bp of the rabbit proTRH mRNA sequence (EU489480), and the D2 probe was complementary to 3230 to 608 bp of the rabbit type II deiodinase gene (EF370408). The fluorescent *in situ *hybridization protocol was adapted from a standard *in situ *hybridization protocol of the TSA™ Biotin System (New England Nuclear, Boston, MA). The antisense RNA probes were digoxin/digoxigenin labeled, diluted in hybridization buffer and hybridized on 10 μm tissue cryosections overnight in a humidified stove at 62°C. After washing, the sections were incubated with an anti-digoxin/digoxigenin horseradish peroxidase-labeled antibody. The probe was amplified using Tyramide Amplification Reagent (TSA™ Biotin System, NEN, Boston, MA), and visualized with streptavidine conjugated to Cy3. Following washes, the sections were mounted in Fluorescent Mounting Medium (DAKO, Glostrup, Denmark) with 4',6-diamidino-2-phenylindole (Sigma-Aldrich, Bornem, Belgium) to counter stain cell nuclei. We analyzed sense-probes as negative controls. As it is not possible to quantitate data obtained from fluorescent *in situ *hybridization with tyramide amplification, we attempted to do isotopic *in situ *hybridizations. This resulted in high background and low signal to noise ratio and results could not be used for analysis.

### RNA isolation and real-time PCR

Gene expression analysis was performed on eight healthy rabbits vs. six prolonged ill animals. RNA was isolated from the total hypothalamic block using the RNeasy midi RNA isolation kit (Qiagen, Venlo, The Netherlands) and quantified by Nanodrop spectrophotometer (ND-1000, Nanodrop Technologies, Wilmington, DE, USA). Samples were treated with DNAse to remove all contaminating genomic DNA. A 1 μg sample of total RNA was reverse-transcribed using random hexamers. All samples were reverse transcribed simultaneously. Reactions lacking reverse transcriptase were also run as a control for genomic DNA contamination.

D2, MCT8, MCT10, OATP1C1, TRα1, TRα2, TRβ1 and TRβ2 mRNA levels were quantified in real time with the ABI PRISM 7700 sequence detector (Applied Biosystems, Foster City, CA) which uses TaqMan chemistry for highly accurate quantization of mRNA levels. Sequences of the primers and probes are given in Table 1. The 10 μl real-time reaction mixture contained 5 μl TaqMan^® ^Fast Universal PCR Master Mix (Applied Biosystems, Foster City, CA), 0.5 μl forward primer (18 μM), 0.5 μl reverse primer (18 μM), 0.5 μl TaqMan probe ([5']6-FAM [3']BHQ-1 labeled) (6 μM), 0.5 μl water and 3 μl cDNA (7.5 ng). Unknown samples were run in duplicate and individual samples with a Ct value standard deviation greater than 0.3 were reanalyzed. Data were analyzed using the comparative Ct method. The rabbit *hypoxanthine guanine phosphoribosyl transferase *(*HRPT*) gene was first cloned and absolute quantification was performed using a standard curve. This showed no significant difference between healthy control and prolonged ill rabbits. *HPRT *was therefore used as an internal control.

**Table 1:** Real-time polymerase chain reaction primers and probes

Gene name [Genbank accession number]		Sequence
ocD2 [GenBank:EF370408]	Forward:	5'-GGACTCCGCTGTGTCTGGAA-3'
	Probe:	5'-CTTGACGCCTACAAACA-3'
	Reverse:	5'-GGCATCCTCGCCCAATTT-3'
ocMCT8 [GenBank:EF420874]	Forward:	5'-CCATGTGGCCTTCTACTTTGC-3'
	Probe:	5'-CCCCCCATCATTGGAGCTGTCATC-3'
	Reverse:	5'-TGCATCAGAGGGACGAAGAAA-3'
ocMCT10 [Genbank:EF489851]	Forward:	5'-TCCGAGCAGAAATCCAATCG-3'
	Probe:	5'-TTGGGACACATCCCGGGCACC-3'
	Reverse:	5'-GGCTCCCATTGCCTTTGAG-3'
ocOATP1C1 [GenBank:EF420875]	Forward:	5'-GATCAGCGGTCTTTGGTTACCT-3'
	Probe:	5'-CTGTTCCTTTCCCTGTTTGCACTGGG-3'
	Reverse:	5'-TGCCACATCCAAGTTTTCACA-3'
ocTRα1 [GenBank:EU489476]	Forward:	5'-GAGTGCCCCACCGAACTCT-3'
	Probe:	5'-TCCCCCACTCTTCCTCGAGGTCTTTG-3'
	Reverse:	5'-CCGCCTGAGGCTTTTAGACTT-3'
ocTRα2 [GenBank:EU489477]	Forward:	5'-AAGTGCAGAGTTCGATTCTGTACAA-3'
	Probe:	5'-CGGGTCACTGGGCGTCCACC-3'
	Reverse:	5'-GAACAACATGCATTCCGAGAAG-3'
ocTRβ1 [GenBank:EU489478]	Forward:	5'-GCGCAGCGCGTTGAA-3'
	Probe:	5'-AACGAACAGTCATCACCACATCTCATCCAG-3'
	Reverse:	5'-GGATGGAGCTCGTCCAAGTG-3'
ocTRβ2 [GenBank:EU489479]	Forward:	5'-GCCATCCTGACTATTTCACTGAAGA-3'
	Probe:	5'-AAGCCTACTTTTTCTCAAGGGCAGTCACCG-3'
	Reverse:	5'-GGGATGTACCCTTTTTTCTGAGAGT-3'
ocHPRT [GenBank:AF020294]	Forward:	5'-TGTAGATTTTATCAGACTGAAGAGCTACTGT-3'
	Probe:	5'-TTTCCAGTTAAGGTTGAGAGATCATCTCCACCGAT-3'
	Reverse:	5'-AAGGAAAGCAAGGTCTGCATTGTT-3'

### Statistical analysis

All statistical analyses were done using StatView software (SAS Institute Inc., Cary, NC, USA). Data were analyzed using one-way analysis of variance tests. Data are presented as mean ± standard deviation. Statistical significance was assumed for a two sided *P *< 0.05.

## Results

**Figure 1:**
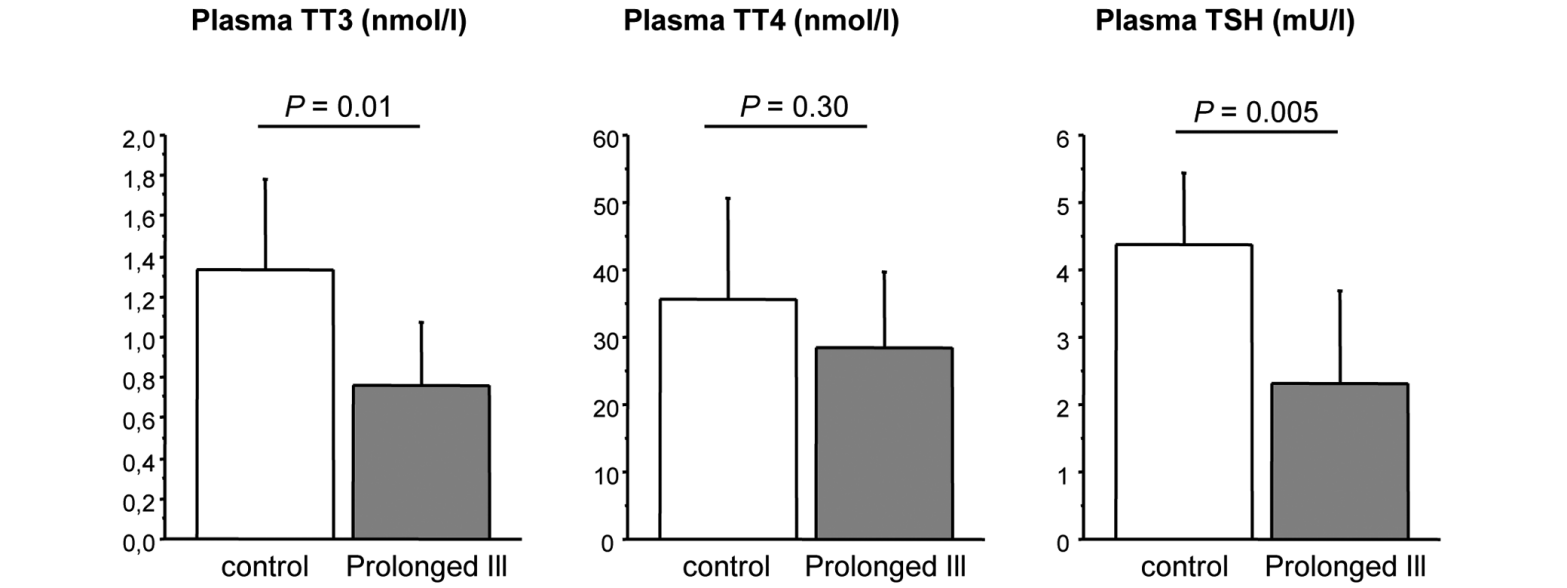
Circulating hormone parameters in healthy control (n = 10) and prolonged ill rabbits (n = 11). Data are expressed as mean ± standard deviation. TT3 = Total T3; TT4 = Total T4; TSH = thyroid stimulating hormone.

As described previously [21], this rabbit model of prolonged critical illness is characterized by low T_3 _plasma concentrations in the face of decreased TSH levels (Figure 1).

**Figure 2:**
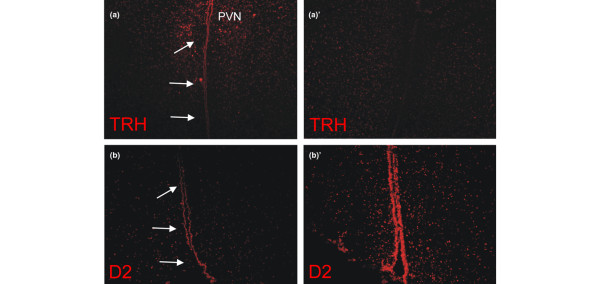
Fluorescence *in situ *hybridization staining for **(a, a')** TRH, and **(b, b')** D2 on frozen sections of the hypothalamus. **(a, b) **Healthy control vs. **(a', b') **prolonged ill animals. **(a, a') **Magnification × 5, **(b, b') **× 10. D2 = type II iodothyronine diodinase; PVN = paraventricular nucleus; TRH = thyrotropin releasing hormone. Arrows represent third ventricle.

TRH is expressed at different sites in the hypothalamus; therefore, measuring TRH expression in homogenates of the total hypothalamic block would not selectively reflect hypophysiotropic TRH expression. We therefore used *in situ *hybridization histochemistry to visualize TRH mRNA expression and observed markedly reduced hybridization signal in the PVN of prolonged ill animals confirming the prolonged critically ill state of our animals. (Figures 2a, a', b and 2b').

**Figure 3:**
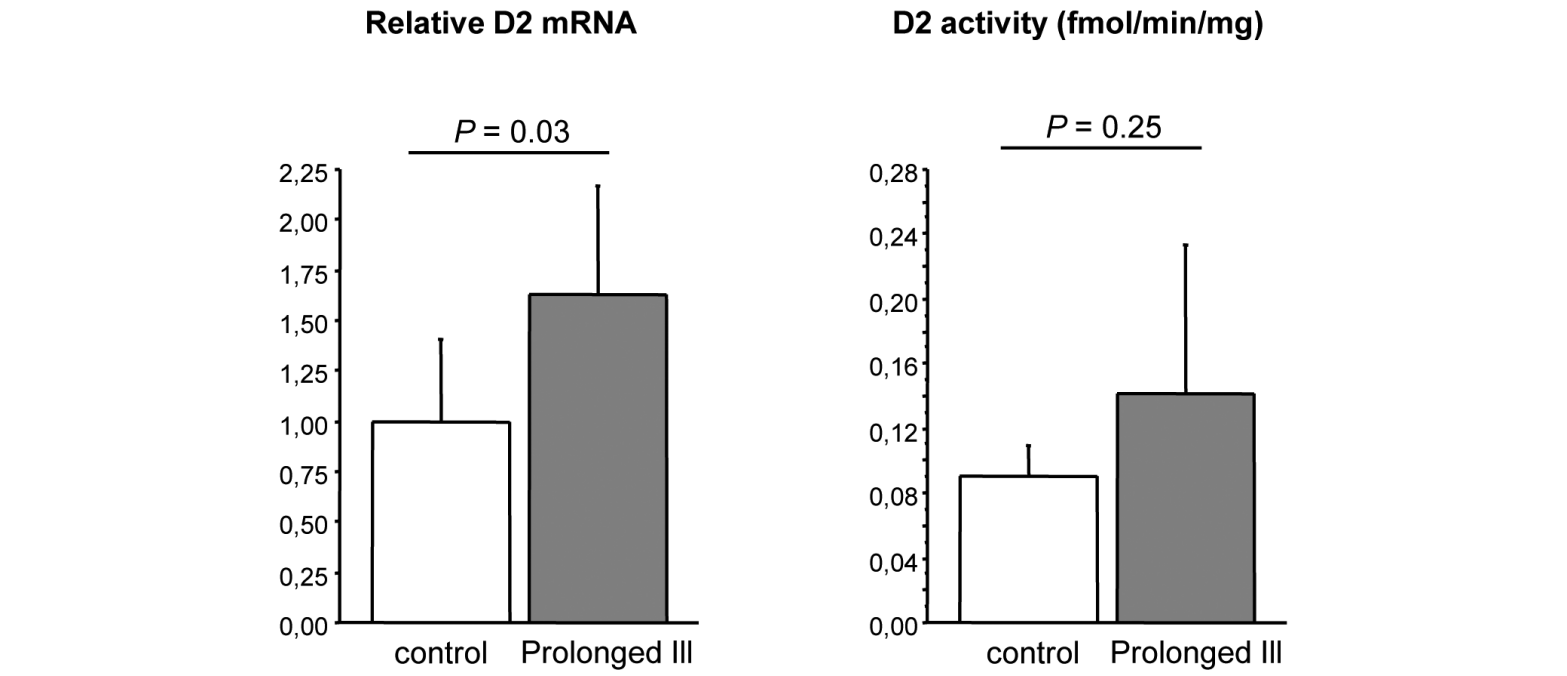
Relative D2 mRNA expression levels and D2 activity measured in the whole hypothalamus of healthy rabbits and prolonged ill rabbits. Healthy rabbits: n = 5 for activity, n = 8 for expression values; prolonged ill rabbits: n = 6 for activity, n = 6 for expression values. Data are expressed as mean ± standard deviation. D2 = type II iodothyronine diodinase.

D2 mRNA expression by *in situ *hybridization showed to be strongly increased in the mediobasal hypothalamus of prolonged ill animals, mostly in the floor and infralateral walls of the third ventricle (Figures 2b, b'). This increase was confirmed by quantitative real-time PCR measurement (*P *= 0.03; Figure 3). D2 activity tended to be increased in sick rabbits but this did not reach significance (prolonged ill rabbits 0.14 ± 0.09 *vs*. healthy controls 0.09 ± 0.02, *P *= 0.25; Figure 3).

Incubation of hypothalamic homogenates with [^125^I]T_4 _as described in Materials and Methods resulted in a 30 to 50% conversion of the substrate to [^125^I]rT_3_. Reverse T_3 _production was inhibited more than 80% by addition of 0.1 μM T_3 _to the incubation mixture, confirming that this represents D3 activity. D3 activity was not significantly different but tended to be higher in chronically ill rabbits (prolonged ill rabbits 1.63 ± 0.45 fmol/min/mg protein *vs. *healthy controls 1.24 ± 0.34; *P *= 0.14).

**Figure 4:**
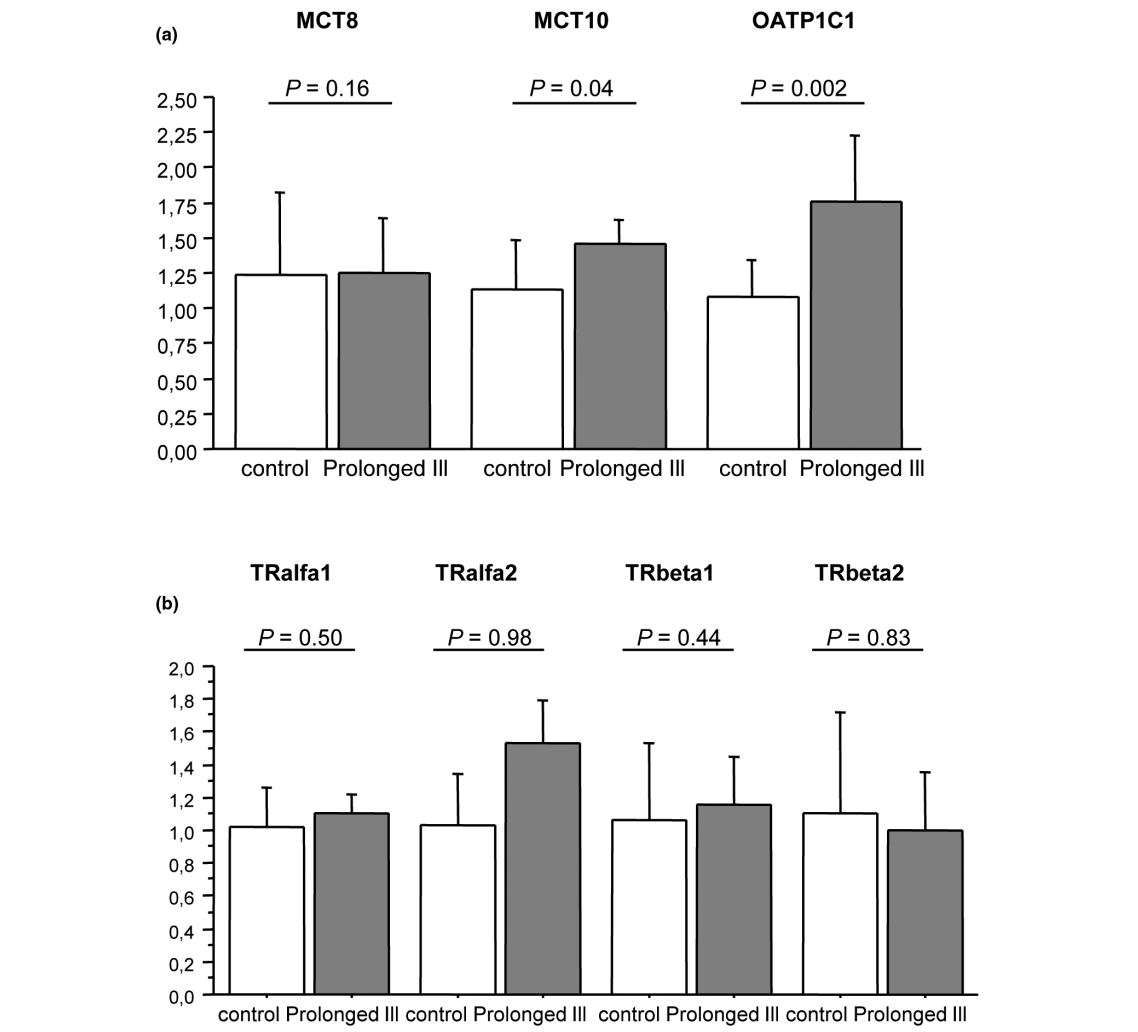
Relative mRNA expression of thyroid hormone transporters and thyroid hormone receptors measured in hypothalamus of healthy control and prolonged ill rabbits. **(a) **Thyroid hormone transporters measured were MCT8, MCT10 and OATP1C1 and **(b) **thyroid hormone receptors measured were TRα1, TRα2, TRβ1 and TRβ2 in hypothalamus of healthy control (n = 8) and prolonged ill rabbits (n = 6). Data are expressed as mean ± standard deviation.

Expression levels of thyroid hormone transporters MCT10 (*P *= 0.04) and OATP1C1 (*P *= 0.002) were significantly increased in the hypothalamus of prolonged ill animals (Figure 4a). There was no change in MCT8 gene expression (Figure 4a).

Hypothalamic TRα1, TRα2, TRβ1 and TRβ2 expression was not significantly different in prolonged ill animals as compared with healthy controls (Figure 4b).

**Figure 5:**
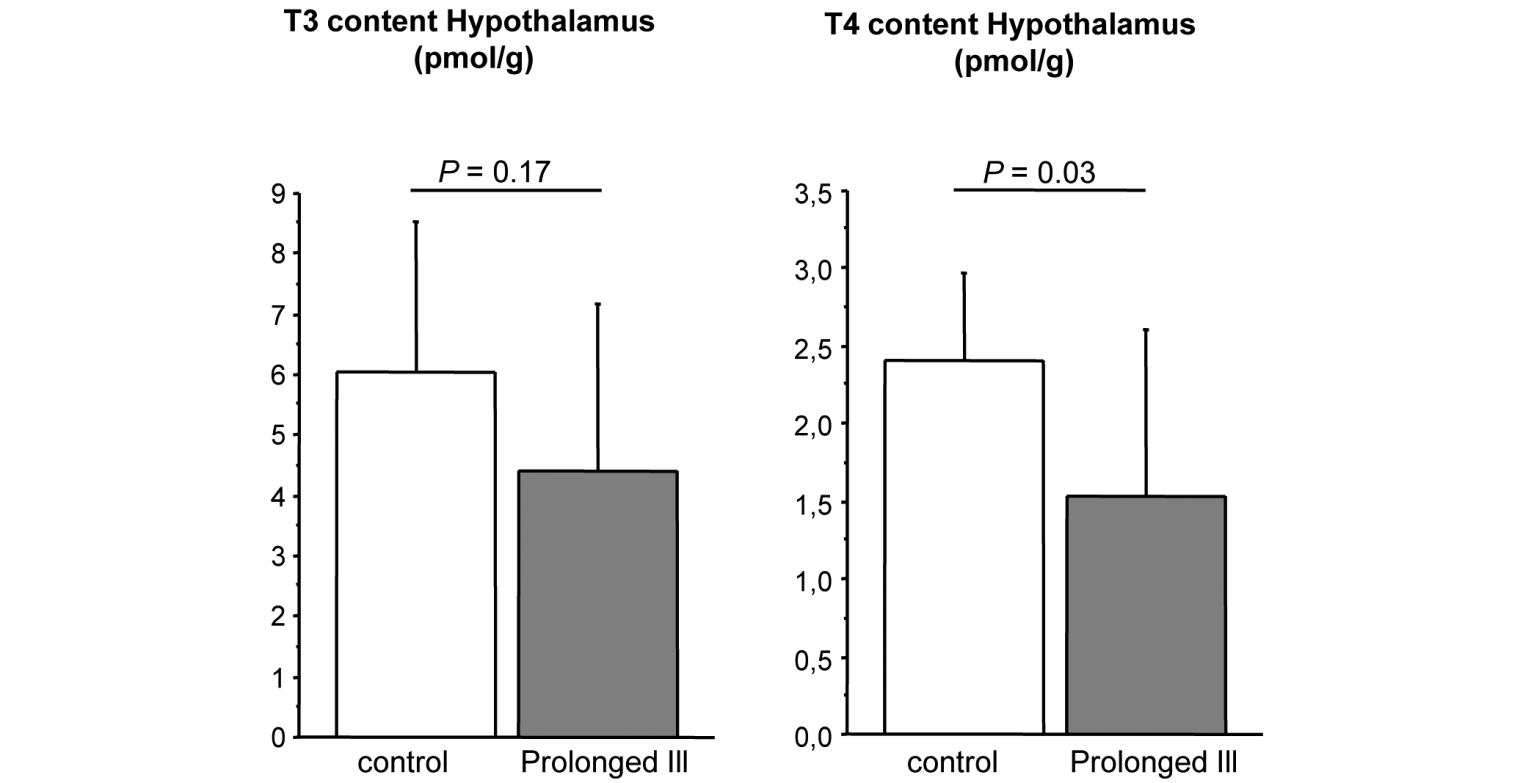
Local thyroid hormone concentrations in hypothalamus of healthy control (n = 10) and prolonged ill rabbits (n = 11). Data are expressed as mean ± standard deviation. TT3 = Total T3; TT4 = Total T4

In prolonged ill rabbits, we measured a 40% reduction in hypothalamic T_4 _content as compared with healthy rabbits (*P *= 0.03, Figure 5). T_3 _content was not significantly different between the two groups, but tended to be lower in the critically ill animals (*P *= 0.17, Figure 5).

## Discussion

Prolonged critical illness is hallmarked by reduced TRH gene expression in the face of low circulating T_3 _levels. In our animal model of prolonged critical illness, we investigated whether reduced TRH could be the result of feedback inhibition exerted by increased local T_3 _levels in the hypothalamus. We found increased D2 mRNA and increased thyroid hormone transporter gene expression (MCT10 and OATP1C1) in prolonged critically ill animals. These changes could lead to increased local T_3 _levels supporting our hypothesis. However, local T_4 _levels in the hypothalamus were lower in the critically ill than in healthy control animals, whereas local T_3 _levels were similar. There was no change at the thyroid hormone receptor level.

By *in situ *hybridization staining we observed an almost complete loss of TRH signal in the PVN of prolonged ill animals. This confirms data from Fliers and colleagues who have clearly shown that TRH is reduced during prolonged critical illness [4].

D2 gene expression was markedly increased in the mediobasal hypothalamus in the prolonged critically ill state as was seen by *in situ *hybridization staining. This was confirmed when we quantified D2 gene expression levels with real-time PCR. We could not measure a significant difference in D2 activity levels although they seemed to have increased in prolonged critically ill animals. It is possible that this is due to a dilution effect because we measured deiodinases activity in the entire hypothalamic block. However, these results are very similar to those observed in hypothyroid animals where D2 gene expression was also found to be moderately increased without an overt increase in D2 activity [7]. In contrast, fasting has shown to evoke a more robust increase in D2 mRNA as well as activity levels [7]. A starvation-induced rise in D2 can be excluded in our model because all animals were fed via the parenteral route which guaranteed uptake of the delivered calories despite the anorexia accompanying illness. As Fekete and Lechan stated, relatively stable levels of D2 activity in the mediobasal hypothalamus is necessary for normal negative feedback regulation of the HPT axis. This allows hypophysiotropic TRH neurons to sense any changes in circulating T_4 _levels [27].

A decrease in D3 activity is another possibility to increase local T_3 _levels. Hypothalamic D3 activity, however, showed an opposite change, because it was not reduced and even tended to be higher in sick animals.

We examined the expression of thyroid hormone transporters because an elevated transport of iodothyronines into the hypothalamus could also contribute to increased local T_3 _levels. MCT8 is expressed by neurons of the PVN, supraoptic, and infundibular nuclei [28] and analysis of two different knockout models showed the importance of MCT8 for thyroid hormone entry into the brain [15,16]. We observed no significant change in MCT8 gene expression during prolonged critical illness in our rabbit model. However, we measured an increase in MCT10 and OATP1C1 mRNA levels. Ramadan and colleagues showed low gene expression levels of MCT10 in total brain RNA extracts [29]. We are the first to show specific hypothalamic MCT10 expression. It was only very recently that MCT10 was shown to be a very active transporter of T_3 _and, to a lesser extent, of T_4 _[19]. The T_4_-specific transporter OATP1C1 was previously shown to be regulated by thyroid hormone [13]. In hypothyroid rats, the expression of OATP1C1 is increased in brain capillaries and, conversely, hyperthyroid rats show decreased expression of this transporter. Regulation of OATP1C1 can thus be an adaptive response to protect hypothalamic thyroid hormone levels against fluctuating plasma levels.

Increased expression of thyroid hormone receptors is another way to increase thyroid hormone activity and thereby reducing TRH expression in the face of normal or low hypothalamic thyroid hormone levels, and expression of thyroid hormone receptor isoforms was shown to be regulated by thyroid hormone status in the hypothalamus [30]. However, our results did not support such a mechanism, because the expressions of TRα1, TRα2, TRβ1 and TRβ2 were unaltered in prolonged critically ill animals as compared with those in healthy controls.

Increased expression of D2 and increased expression of thyroid hormone transporters, as we observed in the prolonged critically ill rabbits, could theoretically lead to increased local T_3 _levels, explaining the suppressed TRH gene expression and the low circulating TSH levels. However, unexpectedly, local T_3 _concentrations were not increased and even tended to be low and hypothalamic T_4 _content was significantly reduced in prolonged ill animals. Data on local levels of thyroid hormones in the hypothalamus during prolonged critical illness are scarce. A study by Arem and colleagues showed that the hypothalamus of patients who died after chronic severe illness contains less than half the concentration of T_3 _as compared with patients who died from an acute trauma [31], which is in line with our data. There are two possible ways to interpret our findings. Because we measured iodothyronine concentrations in the entire hypothalamic block, we cannot exclude a dilution effect. Alternatively, local thyroid hormone content in the hypothalamus could indeed be low during prolonged critical illness. In that case, other mechanisms inferentially are responsible for reducing TRH gene expression during prolonged critical illness and the increased D2 and increased thyroid hormone transporter gene expression levels which we observed could reflect a compensatory response to a local hypothyroid state. Such a compensatory response would be in line with the upregulated D2 expression and activity documented in skeletal muscle of prolonged critically ill patients [32].

Some limitations of our study should be addressed. Our animal model of burn injury-induced critical illness may mirror only part of the complex entity of human critical illness, and thus extrapolating to the human situation or to other illnesses should be done with great caution. Secondly, we were only able to measure gene expression levels of thyroid hormone receptors and transporters. This does not necessarily reflect transporter activity levels.

## Conclusions

In conclusion, although hypothalamic D2 mRNA and gene expression of the thyroid hormone transporters MCT10 and OATP1C1 were increased in our animal model of prolonged critical illness, we failed to detect an increase in local T_3 _levels. This suggests that the reduced hypothalamic TRH expression in our animal model of prolonged critical illness is not necessarily the exclusive result of feedback-inhibition by locally elevated T_3 _levels. Other illness-related factors could be inferred to suppress the TRH gene and the increased expression of thyroid hormone transporters (MCT10 and OATP1C1) and of D2 may reflect a compensatory response to a central hypothyroid state during prolonged critical illness.

## Key messages

• D2 mRNA and expression of thyroid hormone transporters MCT10 and OATP1C1 is increased in the hypothalamus of prolonged critically ill rabbits.

• Hypothalamic T3 and T4 levels are not increased in a rabbit model of prolonged critical illness.

• Reduced TRH gene expression in the context of non-thyroidal illness cannot be explained by negative feedback inhibition by locally elevated T3 levels.

## Abbreviations

bp: base pair; BSA: bovine serum albumin; CV: coefficient of variation; D1: type 1 iodothyronine deiodinase; D2: type 2 iodothyronine deiodinase; D3: type 3 iodothyronine deiodinase; HPRT: hypoxanthine guanine phosphoribosyl transferase; HPT: hypothalamus-pituitary-thyroid; MCT: monocarboxylate transporter; OATP: organic anion transporting polypolypeptide; PCR: polymerase chain reaction; PN: parenteral nutrition; PVN: paraventricular nucleus; RIA: radioimmunoassay; T3: 3,5,3'-triiodothyronine; T4: thyroxine; TR: thyroid hormone receptor; TRH: thyrotropin releasing hormone; TSH: thyroid stimulating hormone, thyrotropin.

## Competing interests

The authors declare that they have no competing interests.

## Authors' contributions

LM carried out the molecular genetic studies, the fluorescence *in situ *hybridizations, and drafted the manuscript. YD, BE, SD, and EJV carried out the rabbit experiments. LL participated in the design of the study and helped to draft the manuscript. VMD carried out the serum analysis and measured thyroid hormone content in the hypothalamus. EF participated in the design of the study and assisted with the *in situ *hybridizations. TJV assisted with the cloning of all the rabbit genes. GVdB conceived of the study, and participated in its design and coordination and helped to draft the manuscript. All authors read and approved the final manuscript.
